# Nanocarrier of α-Tocopheryl Succinate Based on a Copolymer Derivative of (4,7-dichloroquinolin-2-yl)methanol and Its Cytotoxicity against a Breast Cancer Cell Line

**DOI:** 10.3390/polym15224342

**Published:** 2023-11-07

**Authors:** Hernán Valle, Raquel Palao-Suay, María Rosa Aguilar, Tulio A. Lerma, Manuel Palencia, Ramalinga Viswanathan Mangalaraja, Leonardo Guzmán, Dairo Pérez Sotelo, José Becerra

**Affiliations:** 1 Chemistry Department, Faculty of Basic Sciences, University of Córdoba, Montería 230002, Colombia; 2 Laboratory of Chemistry of Natural Products, Department of Botany, Faculty of Natural and Oceanographic Sciences, University of Concepción, Casilla 160-C, Concepción 4070386, Chile; jbecerra@udec.cl; 3 Group of Biomaterials, Department of Polymeric Nanomaterials and Biomaterials, Institute of Polymer Science and Technology, Spanish National Research Council (ICTP-CSIC), 28006 Madrid, Spain; rpsuay@ictp.csic.es (R.P.-S.); mraguilar@ictp.csic.es (M.R.A.); 4 Networking Biomedical Research Centre in Bioengineering, Biomaterials and Nanomedicine, CIBER-BBN, 28029 Madrid, Spain; 5 Research Group in Science with Technological Applications (GI-CAT), Department of Chemistry, Faculty of Natural and Exact Science, University of Valle, Cali 760042, Colombia; 6 Mindtech Research Group (Mindtech-RG), Mindtech s.a.s., Barranquilla 080006, Colombia; 7 Faculty of Engineering and Sciences, Adolfo Ibáñez University, Diagonal Las Torres 2640, Peñalolén, Santiago 7941169, Chile; 8 Laboratory of Molecular Neurobiology, Department of Physiology, Faculty of Biological Sciences, University of Concepción, Casilla 160-C, Concepción 4070386, Chile; joseguzman@udec.cl

**Keywords:** biomedical applications, copolymers, nanoparticles, radical polymerization, self-assembly, anticancer

## Abstract

In order to improve the water solubility and, therefore, bioavailability and therapeutic activity of anticancer hydrophobic drug α-tocopherol succinate (α-TOS), in this work, copolymers were synthesized via free radicals from QMES (1-[4,7-dichloroquinolin-2-ylmethyl]-4-methacryloyloxyethyl succinate) and VP (N-vinyl-2-pirrolidone) using different molar ratios, and were used to nanoencapsulate and deliver α-TOS into cancer cells MCF-7. QMES monomer was chosen because the QMES pendant group in the polymer tends to hydrolyze to form free 4,7-dichloro-2-quinolinemethanol (QOH), which also, like α-TOS, exhibit anti-proliferative effects on cancerous cells. From the QMES-VP 30:70 (QMES-30) and 40:60 (QMES-40) copolymers obtained, it was possible to prepare aqueous suspensions of empty nanoparticles (NPs) loaded with α-TOS by nanoprecipitation. The diameter and encapsulation efficiency (%EE) of the QMES-30 NPs loaded with α-TOS were 128.6 nm and 52%; while for the QMES-40 NPs loaded with α-TOS, they were 148.8 nm and 65%. The results of the AlamarBlue assay at 72 h of treatment show that empty QMES-30 NPs (without α-TOS) produced a marked cytotoxic effect on MCF-7 breast cancer cells, corresponding to an IC_50_ value of 0.043 mg mL^−1^, and importantly, they did not exhibit cytotoxicity against healthy HUVEC cells. Furthermore, NP-QMES-40 loaded with α-TOS were cytotoxic with an IC_50_ value of 0.076 mg mL^−1^, demonstrating a progressive release of α-TOS; however, the latter nanoparticles were also cytotoxic to healthy cells in the range of the assayed concentrations. These results contribute to the search for a new polymeric nanocarrier of QOH, α-TOS or other hydrophobic drugs for the treatment of cancer or others diseases treatable with these drugs.

## 1. Introduction

In 2020, there were approximately 10 million deaths from cancer and 20 million cases diagnosed worldwide, which will increase to 30 million in 2040, according to WHO predictions. Lung, prostate, colon, stomach, liver, breast, cervical and thyroid cancers are the most common [1]. Currently, many research groups address the problem of cancer with drug combination therapies instead of monotherapy. The purpose of combination therapy is to maximize the efficacy of anticancer drugs by administering them along with another or others that serve(s) as adjuvant(s), which results in synergistic effects [2].

Regarding this strategy, it is worth noting the growing use of therapies that combine a conventional anticancer drug (e.g., doxorubicin, paclitaxel, cisplatin, mitomycin C, bortezomib, temozolomide) with another drug derived from quinoline, such as: hydroxychloroquine, chloroquine [3,4,5], amodiaquine [6,7] (antimalarials), oxyquinoline, iodoquinol [8], clioquinol and nitroxoline [8,9,10]. Some anticancer action mechanisms that have been associated with quinoline molecules include: cell cycle arrest, angiogenesis inhibition, apoptosis, disruption of cell migration, autophagy inhibition and the modulation of nuclear receptor responsiveness. As a result, these types of molecules are often used to treat the most common cancers mentioned above, in addition to those that affect: the lymphatic system, bone marrow, kidneys, ovaries, skin and mouth [11].

However, a common aspect of monotherapy and combination therapy of anticancer drugs is the use of cytotoxic molecules of low molar mass which are poorly soluble in physiological medium and act by destroying the cell division capacity of both neoplastic cells and normal cells. These hydrophobic molecules remain in the circulatory system for a very short time, do not efficiently cross biological membranes and are rapidly absorbed by macrophages. Due to the poor selectivity of these molecules towards cancer cells, their therapeutic use generates harmful side effects that require dose reduction and delay or the interruption of treatment [12].

Fortunately, since the mid-1990s, many cancer researchers have managed to reduce the toxicity of anticancer molecules, using nanomaterials for their encapsulation and subsequent controlled release in tumor cells, without affecting healthy cells [13]. One of these nanomaterials are spherical particles that are ~100 to 200 nm in diameter, produced from biocompatible and biodegradable polymers (with masses close to 30 kDa) [13,14,15] and capable of incorporating anticancer molecules through covalent or non-covalent binding (physical entrapping). When the binding is covalent, the NPs are called “polymeric prodrug nanoparticles” (PDNP) [16,17,18,19], which allow a more controlled release of the drug than NPs with non-covalent binding [20]. In addition, PDNPs can be made of natural polymers or synthetic polymers, but the latter have a lower risk of inducing infections and immunogenicity than those based on animal- or plant-derived polymers [21].

The selectivity of PDNPs towards tumor cells is due to the higher permeability, lower pH and absence of functional lymphatic drainage in tumor tissue, compared to healthy cell tissue. This favors the accumulation, prolonged permanence time and acid and/or enzymatic hydrolysis (e.g., with esterases) of PDNPs inside tumor cells, and consequently, the controlled release of the drug in the cytosol [13,22]. This passive phenomenon is known as the enhanced permeability and retention effect (EPR effect).

Importantly, some PDNPs that incorporate a second drug by physical entrapping, in addition to the drug covalently linked to the polymer, have been effective in antitumor drug combination therapy [16,19,23,24,25]. However, after an exhaustive literature search, no studies were found where conventional anticancer drugs, such as doxorubicin (DOX), paclitaxel (PTX) or α-tocopherol succinate (α-TOS), have been encapsulated (physically entrapped) by PDNP derivatives of anticancer quinoline molecules. In order to verify the effectiveness of this strategy, the present research paper describes the synthesis, by conventional radical polymerization, of a new vinyl copolymer from VP (N-vinyl-2-pyrrolidone) and QMES (1-[4,7-dichloroquinolin-2-ylmethyl]-4-methacryloyloxyethyl succinate) and also evaluates if the copolymer can: (1) form NPs in aqueous medium and encapsulate the α-TOS drug using the nanoprecipitation technique; and (2) inhibit the viability of MCF-7 (breast adenocarcinoma) cells in the AlamarBlue assay.

The importance of the QMES units in the poly(QMES-co-VP) copolymer lies in that they contain the anticancer molecule 4,7-dichloro-2-quinolinemethanol (QOH) that is linked by ester bond to the rest of the polymer. This quinoline molecule has antiproliferative activity on Caco-2 (colon adenocarcinoma) and HTB-129 (breast carcinoma) cells, being 14 and 2 times more potent compared to the drug cisplatin, but also 1.5 times more toxic to normal cells [26]. QOH can be released from the copolymer by acid and/or enzymatic hydrolysis inside cancer cells, generating a possible combined effect with nanoencapsulated α-TOS. On the other hand, α-TOS is an interesting anticancer drug model to nanoencapsulate with poly(QMES-co-VP), due to the fact that: (1) it is less expensive to produce for the pharmaceutical industry than DOX, PTX and other conventional antineoplastic agents, since it is synthesized by a single step from the easily available α-tocopherol (vitamin E); and (2) it is also hydrophobic, poorly selective towards diseased cells and causes negative side effects in cancer patients [27,28].

**Figure 1:**
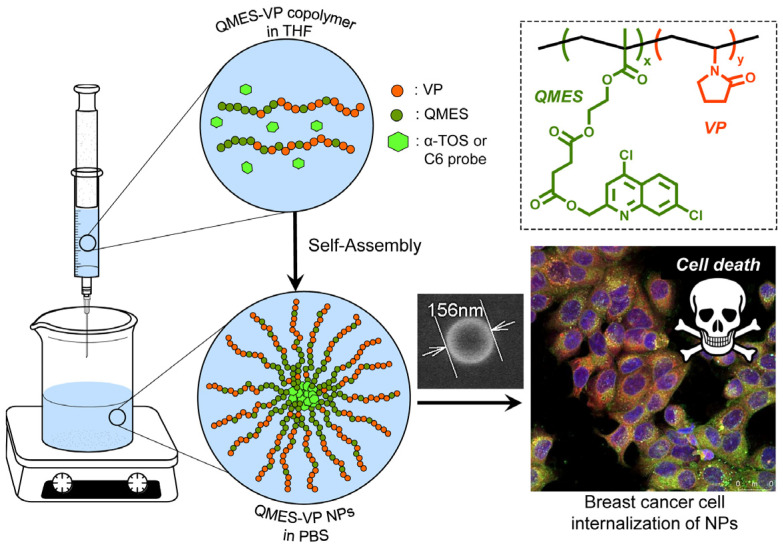
Schematic representation illustrating the preparation of α-TOS loaded QMES-VP copolymer nanoparticles, chemical structure of copolymer and nanoparticles internalization in breast cancer cell.

As a result of the present study, α-TOS-loaded polymeric NPs were obtained using the QMES-40 copolymer, of which the IC_50_ value against MCF-7 cells was 0.076 mg mL^−1^. The foregoing suggests that this new QOH-based PDNP system has potential use in anticancer therapy as a platform for the controlled and targeted delivery of QOH along with α-TOS or other hydrophobic drugs. An overview of this research is presented in Figure 1.

## 2. Experimental

### 2.1. Materials and Reagents

The following reagents and solvents were purchased from commercial sources and used without further purification to synthesize and purify the QMES monomer: 4-dimethylaminopyridine (DMAP, Sigma, Burlington, MA, USA), mono-(2-(methacryloyloxy)ethyl) succinate (MES), dichloromethane (DCM), N,N-dimethylformamide anhydrous (DMF), 1-ethyl-3-(3′-dimethylaminopropyl)-carbodiimide (EDC), which were manufactured by Sigma in St. Louis, MO, USA; sodium bicarbonate (NaHCO_3_), sodium sulfate anhydrous (Na_2_SO_4_), aluminum oxide 90 active neutral (70–230 mesh), n-hexane and ethyl acetate (AcOEt), which were manufactured by Merck in Darmstadt, Germany. 4,7-dichloro-2-quinolinemethanol (QOH) was previously synthesized and purified according to the protocol of Valle et al. [29]. Silica gel 60 F254 chromatoplates (Merck, Darmstadt, Germany) and a Camag^®^ uv lamp 4 dual wavelength 254/366 nm (Camag, Muttenz, Switzerland) were used for analytical thin layer chromatography (TLC).

N-vinylpyrrolidone (VP, Merck, Darmstadt, Germany) and 1,4-dioxane (Sigma, St. Louis, MO, USA) purified by vacuum distillation, 2,2′-azobisisobutyronitrile (AIBN, Merck, Darmstadt, Germany) recrystallized from methanol and Spectra/Por dialysis membranes of 3.5 kDa MWCO (Spectrumlab, Rancho Dominguez, CA, USA) were used to synthesize and purify the QMES-VP copolymers and homopolymers.

### 2.2. Spectroscopic Characterization

All proton NMR (nuclear magnetic resonance) spectra were obtained at 25 °C on a Bruker AscendTM 400 MHz NMR spectrometer with chloroform-*d* (CDCl_3_, 98%; Sigma-Aldrich, St. Louis, MO, USA) or dimethyl sulfoxide-*d*_6_ (MagniSolve, 99.96%, Zurich, Switzerland) as the solvent. The samples were dissolved in deuterated solvent at a concentration of 40 mg mL^−1^. Chemical shifts were recorded in δ values in parts per million (ppm) relative to the appropriate residual solvent peaks (CDCl_3_ or DMSO-*d*_6_), and coupling constants (J) were reported in Hertz (Hz). Abbreviations used in the description of ^1^H NMR data are as follows: (s) singlet, (d) doublet, (dd) double doublet, (ddd) double doublet of doublets and (m) multiplet. MestReNova software (v. 6.0.2, Mestrelab Research, Santiago de Compostela, Spain) was used to normalize and integrate the signals of the spectra. Additionally, the chemical composition of the different copolymers were quantitatively estimated using the normalized signal areas of comonomers in the copolymer spectrum.

### 2.3. Synthesis of QMES

**Scheme 1:**
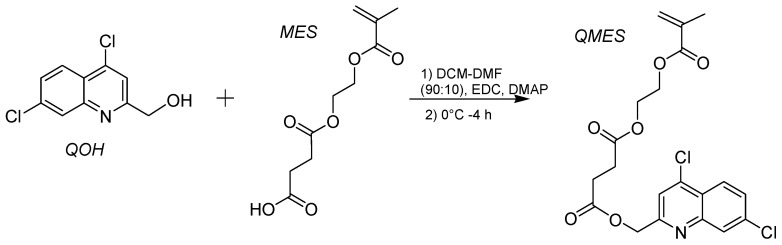
Synthesis of the QMES monomer.

The esterification reaction between 2-(methacryloyloxy)ethyl monosuccinate (MES) carboxylic acid and QOH alcohol was carried out, following a modified version of the Park et al. [30] and Yearick et al. [31] methodology. The chemical reaction is shown in Scheme 1.

An oven-dried 1000 mL three-necked round bottom flask, equipped with a magnetic stir bar, was charged with: 5.67 g QOH (1.0 eq), 6.76 mL MES (1.4 eq), 0.90 g of DMAP (0.3 eq) and 600 mL of a dichloromethane–DMF mixture (90:10).

The round bottom flask was sealed with a rubber septum, purged with nitrogen, magnetically stirred and cooled to 0 °C. Under a nitrogen atmosphere, EDC (7.09 g, 1.5 eq) in dichloromethane (100 mL) was added through the septum, using a syringe. Cooling was stopped after 4 h, and stirring was continued for a further 20 h.

The reaction mixture was transferred to a 2000 mL separatory funnel where it was extracted with a saturated NaHCO_3_ aqueous solution (3 × 500 mL), and then with 5% *w*/*v* LiCl aqueous solution (3 × 500 mL). A total of 600 mL of organic phase was obtained, which was dried over Na_2_SO_4_ for 24 h, filtered through Whatman No. 2 paper and concentrated under vacuum with a rotary evaporator (35 °C).

The obtained viscous residue was purified by flash chromatography using neutral Al_2_O_3_ and hexane–ethyl acetate (Hex-AcOEt) 1:1, as the stationary and mobile phases, respectively. The eluate was concentrated under reduced pressure at 35 °C by rotary evaporation (until all the solvent was removed) and allowed to cool to room temperature. Finally, the resulting solid product, QMES, was vacuum dried over phosphorus pentoxide in a desiccator.

### 2.4. Synthesis of Poly(QMES-co-VP) Copolymers

**Scheme 2:**
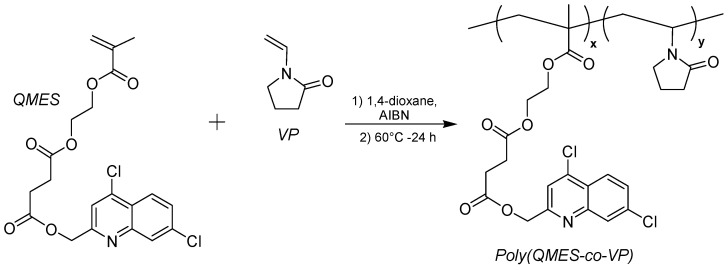
Synthesis of the poly(QMES-*co*-VP) copolymers.

A series of poly(QMES-co-VP) copolymers were synthetized by conventional radical polymerization of QMES and VP using 1,4-dioxane as a solvent and a monomer feed ratio of: 20:80, 30:70, 50:50, 70:30 and 80:20 mol% [QMES]:[VP]. The homopolymers of both monomers were also prepared (PQMES and PVP). The total monomer concentration was of 0.11 molar, and AIBN was used at 5 mol% as a radical initiator (based on the total comonomer concentration). The polymerizations were carried out at 60 °C for 24 h. The reaction products were dialyzed against 2 L of distilled water for 48 h using a membrane with a molecular weight cutoff of 3.5 kDa, and, finally, they were lyophilized. The polymerization reaction is shown in Scheme 2.

### 2.5. Gel Permeation Chromatography (GPC)

QMES-based copolymer apparent average molecular weight (Mn and Mw) and polydispersity index (Ð) were determined by GPC, using a HPLC PerkinElmer series 200 equipment (PerkinElmer, Waltham, MA, USA) with an Isocratic LC pump 250 coupled to a refraction index detector. For this purpose, three polystyrene–divinylbenzene columns (Waters Styragel^®^ HR, Waters Corporation, Milford, MA, USA) were used as a solid phase. The eluent was degassed chromatographic-grade dimethylformamide (DMF, 0.7 mL/min, Scharlau, Barcelona, Spain) with LiBr (0.1% *w*/*v*, Sigma, St. Louis, MO, USA) at a temperature of 70 °C. The calibration curve was obtained using monodisperse polystyrene standards (Agilent Technologies, Santa Clara, CA, USA) with molecular weights between 2.93 and 3039 kDa. Finally, the data were analyzed using the PerkinElmer LC solution program (Waltham, MA, USA).

### 2.6. Differential Scanning Calorimetry

Glass transition temperatures (T_g_) were measured by differential scanning calorimetry (DSC) on a Discovery DSC25 (TA Instruments, New Castle, DE, USA) equipment. The samples (5–10 mg) were placed in Tzero aluminum hermetic pans and heated from −20 to 190 °C at a constant rate of 20 °C min^−1^. Measurements were analyzed using TRIOS Software (v. 4.0, TA Instruments, New Castle, DE, USA). The midpoint of the heat capacity transition was taken as the T_g_.

### 2.7. Thermogravimetric Analysis (TGA)

The thermal stability of the copolymers (~5 mg) was analyzed by thermogravimetric analysis using a TGA Q500 (TA Instruments, New Castle, DE, USA) under a nitrogen atmosphere (50 mL min^−1^) at 10 °C min^−1^ heating rate from 25 to 600 °C. Both the onset and weight loss values were calculated using TA Instruments Universal Analysis Software (v. 4.5A).

### 2.8. Preparation and Characterization of Copolymer Nanoparticles by Nanoprecipitation [14,32]

To obtain unloaded NPs, copolymer solutions were prepared in THF:DMF (7:3) with a 5 mg mL^−1^ concentration. A total of 2.5 mL of each solution was added dropwise to 10 mL of PBS (phosphate buffered solution, pH 7.4), i.e., in 1:4 (*v*/*v*) proportion, under constant magnetic stirring, and then was dialyzed against PBS for 24 h (with a membrane of 3.5 kDa molecular weight cutoff) to remove the organic solvent. The NPs’ suspensions that did not generate visible precipitates after three days at room temperature (most stable ones), and with an average diameter between 100 and 200 nm, were selected to encapsulate the α-TOS anticancer drug. The average diameter, size distribution and polydispersity of the NPs suspensions were determined by dynamic light scattering technique (DLS) using a Nanosizer NanoZS equipment (Malvern Instruments, Malvern, UK). The zeta potential of the NPs was measured by electrophoretic mobility experiments using the same NanoZS equipment. Scanning electron microscopy (SEM) was used for the morphological characterization of the NPs at a NP concentration of 0.01 mg mL^−1^ using a Hitachi SU8000 TED cold-emission field emission SEM microscope (Hitachi, Tokyo, Japan) working at an accelerating voltage 30 kV.

In order to encapsulate α-tocopherol succinate (α-TOS, Sigma-Aldrich, St. Louis, MO, USA) or coumarin 6 (c6, Sigma-Aldrich, Milwaukee, WI, USA) in the selected NPs, such NP suspension was prepared again, but this time starting with the addition of the drugs (10% *w*/*w* of α-TOS or 1% of c6) to the dissolved copolymer in THF:DMF (7:3) (percentage of the drug weight based on both solutes), and then continuing with the same procedure as described in the above paragraph, until obtaining the respective drug-loaded NPs suspended in PBS pH 7.4 (organic solvent-free and without non-encapsulated drugs).

### 2.9. Determination of Encapsulation Efficiency (EE) of α-TOS

α-TOS loaded nanoparticles were freeze dried and an amorphous powder was obtained with a yield higher than 90%. The powder was dissolved in ethanol and stirred in a glass vial sealed for 24 h to ensure NP disaggregation and drug release. Then, samples were centrifuged at 8000 rpm, and the supernatant was analyzed by UV (λ_abs_ = 285 nm, Figure S1, Supplementary Materials) using a NanoDrop™ Onec Microvolume UV-Vis Spectrophotometer (Thermo Scientific™, Waltham, MA, USA). The unknown α-TOS solution concentration was calculated by substituting the absorbance value in the calibration curve equation (extinction coefficient = 2.050), previously obtained with a series of standard solutions of α-TOS in ethanol with concentrations ranging from 1 to 0.001 mg mL^−1^. The encapsulation efficiency (%*EE*) of α-TOS was defined as the ratio between the experimentally measured α-TOS concentration (mg mL^−1^) by spectrophotometry, [*D*]_exp_ (i.e., α-TOS encapsulated in the nanoparticle core) and the concentration established at the start of nanoprecipitation, [*D*]_0_. The equation used was the following:

$$\%EE=\frac{{\left[D\right]}_{exp}}{{\left[D\right]}_{0}}\times 100$$

### 2.10. Cell Culture and Biological Reagents

Dulbecco’s modified Eagle’s medium (DMEM, Sigma-Aldrich, St. Louis, MO, USA), fetal bovine serum (FBS, Gibco), L-glutamine (Sigma-Aldrich, St. Louis, MO, USA), penicillin/streptomycin (P/S, Sigma-Aldrich, St. Louis, MO, USA), glutamax I (Life Technologies, Waltham, MA, USA), heparin (Sigma-Aldrich, St. Louis, MO, USA), no phenol red DMEM (Gibco-Thermo Fisher Scientific, Lafayette, CO, USA), AlamarBlue^®^ (Invitrogen, Eugene, OR, USA) and Trypsin-EDTA solution (Sigma-Aldrich, St. Louis MO, USA) were used for the cellular assays.

### 2.11. Cytotoxicity Assay on MCF-7 Cells and HUVEC Cells

NP toxicity was assessed using human mammary adenocarcinoma cells (MCF-7, ECACC) and human umbilical vein endothelial cells (HUVEC, donated by the Institute of Pathology, Universitätsmedizin, Mainz, Germany). On one hand, MCF-7 cells were grown in Dulbecco’s modified Eagle’s medium (DMEM), supplemented with 10% FBS, 2% L-glutamine and 1%P/S. On the other hand, HUVEC cells were cultured in M199 medium (Sigma-Aldrich) supplemented with 20% FBS, 1% P/S, 1% glutamax I, 25 μg/mL heparin and 25 μg/mL endothelial cell growth supplement (Becton Dickinson). Both cell lines were maintained at 37 °C in a humidified atmosphere of 5% CO_2_.

NP suspensions were sterilized by filtering through 0.22 μm polyethersulfone membranes (Millex-GP PES Millipore Express, Merck, Darmstadt, Germany) and diluted with PBS to obtain different NP concentrations (0.5, 0.25, 0.125, 0.063 and 0.031 mg mL^−1^). Cell viability was quantified using the AlamarBlue assay. For this purpose, MCF-7 (6000 cells/well) and HUVEC cells (5000 cells/well) were seeded into 96-well culture plates and incubated for 24 h. The culture medium was then replaced with a fresh one with the NPs (1:1). After each time (24 and 72 h), the NPs were removed, the cells were washed with PBS and treated with a 10% AlamarBlue^®^ solution in phenol red-free DMEM and incubated for 3 h. After this time, fluorescence was quantified at an excitation/emission of 590/530 nm using a fluorescence microplate reader (Biotek Synergy HT spectrophotometer, BioTek Instruments, Winooski, VT, USA). For each time, cells treated with PBS were used as a control (M + PBS). Experiments were performed using eight or six replicates per formulation.

### 2.12. c6 Loaded NPs Cellular Uptake and Intracellular Location

Fluorescent c6 loaded NPs were used to study the cellular internalization of the NPs. MCF-7 were seeded at 90,000 cells/mL into 8-well plates (1µ-Slide 8 well iBiTreat, Ibidi, Gräfelfing, Germany). After 24 h, the medium was replaced by a mixture 1:1 (*v*:*v*) of NPs (0.5 mg mL^−1^) and DMEM and incubated overnight. After this incubation time, NPs were removed and cells were washed twice with PBS. Additionally, to track the intracellular location of the nanoparticles, cells were stained with 1 µM MitoTracker^®^ Red CMXRos and 0.75 µM LysoTracker^®^ DND99 (Invitrogen, Carlsbad, CA, USA) for 60 min and then washed three times with PBS. Finally, cells were fixed with 3.7% formaldehyde for 20 min at room temperature. Cells were observed using LEICA TCS SP8 STED 3X Confocal Laser Microscope (Leica, Wetzlar, Germany).

### 2.13. Statistical Analysis

The cell viability results were expressed as a percentage with respect to the control group (set as 100%) and presented as mean ± standard deviation. In cases where cell viabilities were not normally distributed across treatment groups (the six NP concentrations, including the control one), non-parametric Kruskal–Wallis one-way analysis of variance on ranks (ANOVA on ranks) and a post-hoc pairwise comparison test (automatically selected by SPSS software) with the Bonferroni adjustment were used to compare the differences between groups. On the other hand, for datasets that were normally distributed, a one-way ANOVA and an LSD multiple-comparison test were used to indicate the differences in cell viabilities among treatments groups. Statistical analyses were performed using SPSS software (version 29.0.1.0; IBM SPSS Statistics, Chicago, IL, USA) or Statgraphics Centurion XVI software (version 16.1.03, StatPoint Technologies, Inc., Warrenton, VA, USA).

## 3. Results and Discussion

### 3.1. Synthesis and ^1^H NMR Analysis of the QMES Monomer

A total of 8.3 g of QMES was obtained as greenish-white crystals, and its purity and molecular formula were confirmed by TLC and ^1^H NMR. The reaction yield was 75%. The TLC silica plates showed only one spot with a Rf value of 0.77 (using hexane-AcOEt 1:1 as an eluent) and the absence of spots from the starting reagents.

**Figure 2:**
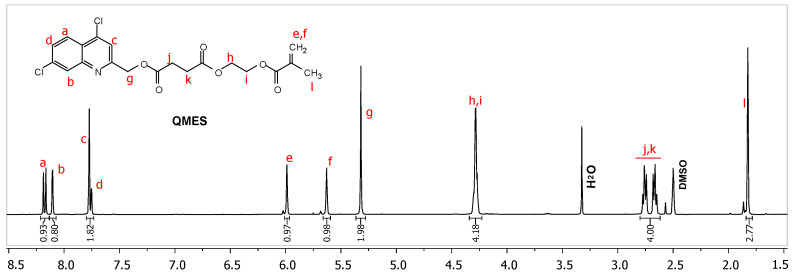
^1^H NMR spectrum of QMES in DMSO-*d*_6_.

In the ^1^H NMR spectrum shown in Figure 2, the characteristic signals of QMES were observed without evidence of contamination. The presence of the vinylic protons ***e*** and ***f*** was inferred from the signals at 5.99 ppm (s) and 5.63 ppm (m), respectively. The signals appearing between 7.7 and 8.2 ppm were attributed to the four aromatic protons: ***a***, ***b***, ***c*** and ***d***. The ***d*** proton at δ 7.76 (dd, J = 8.7, 2.1 Hz) showed an ortho-coupling to the ***a*** proton at δ 8.18 (d, J = 9.0 Hz) and a meta-coupling to the ***b*** proton at 8.11 (d, J = 1.9 Hz), which allowed the locating of a chlorine substituent between the ***b*** and ***d*** protons of the benzenoid part of the quinoline ring. Singlets at δ 7.77 and δ 5.32 ppm were assigned to the ***c*** aromatic proton and the oxymethylene ***g*** proton pair of the pyridine part of the quinoline ring, respectively. The ***j***–***k*** signals at δ 2.71 ppm (ddd) corresponds to the four protons of the succinyl group. The multiplet that appears at δ 4.29 ppm was attributed to the ***h***–***i*** protons of the ethylenedioxy group, and finally, the ***l*** signal at δ 1.83 ppm (s) corresponds to the only methyl group of the molecule.

### 3.2. Synthesis and ^1^H NMR Analysis of QMES-VP Copolymers

**Figure 3:**
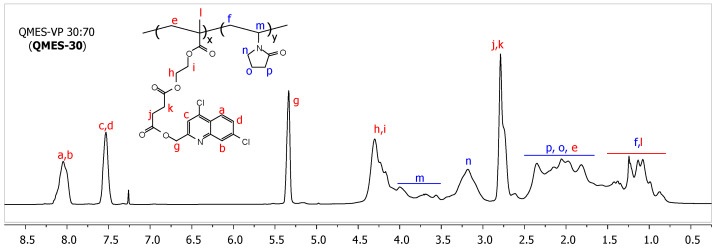
^1^H NMR spectrum of poly(QMES-co-VP) 30:70 in CDCl_3_ (composition of comonomers in the copolymer).

All the copolymers were successfully synthesized (see yields in Table 1), they had the appearance of a whitish powder and their NMR spectra were similar to each other, although with differences in signal intensity, depending on the composition of the comonomers used. As an example, the ^1^H NMR spectrum of the QMES-30 copolymer is shown in Figure 3. The absence of a residual monomer in all samples was verified (successful purification by dialysis against water). The spectral region between 0.5 and 4.5 ppm is a complex superposition of signals attributed to both monomeric residues. The signals ***h***, ***i***, ***j***, ***k*** and ***e***, indicated in the spectrum, were assigned to the ethylenedioxy (***h***, ***i***), succinyl (***j***, ***k***), methyl (***l***) and methylene (***e***) groups of the QMES monomeric unit; while the signals ***m***, ***n***, ***o***, ***p*** and ***f*** correspond to all the protons of the VP monomeric unit, that is: (1) the methine and methylene bound to the nitrogen atom of the pyrrolidone ring (***m*** and ***n***, respectively), (2) the most shielded CH_2_ groups of this ring (signals ***o***, ***p***) and (3) the methylene group of the polymer main chain (***f***). A second region of the spectrum, between 7 and 8.5 ppm, contains the signals ***a***, ***b***, ***c*** and ***d***, corresponding to the aromatic protons of the quinoline ring. Finally, the signal at 5.34 ppm (***g***) is due to -O-CH_2_- moiety bonded directly to the quinoline ring.

The molar fractions of the QMES and VP monomers in each of the synthesized copolymers were calculated using the signal at 5.34 ppm and the group of signals between 0.5 and 4.5 ppm. These signals were integrated using the MestReNova software (version 6.0.2), and the values of the integrated areas were substituted in the following system of equations:

(1) $$F_{QMES}=\frac{Q}{V+Q}$$

(2) $$F_{VP}=\frac{V}{V+Q}$$

(3) $$Q=\frac{{(I}_{5.34\;ppm})}{2}$$

(4) $$V=\frac{{(I}_{0.5-4.5\;ppm\;})-13Q}{9}$$

where Q corresponds to the contribution of one QMES proton to the integrated area of the signal at 5.34 ppm (**I**_5.34 ppm_); V is the contribution of one VP proton to the integrated area of the signal between 0.5 and 4.5 ppm (**I**_0.5–4.5 ppm_); while F_QMES_ and F_VP_ are the molar fractions of the QMES and VP monomers in the copolymer, respectively. Table 1 shows the QMES molar fractions of each copolymer (F).

### 3.3. DSC Analysis of QMES-VP Copolymers and Homopolymers

**Figure 4:**
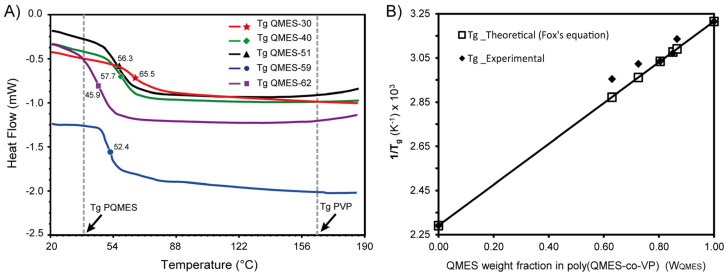
(**A**) DSC Thermograms of poly(QMES-co-VP) copolymer series, and (**B**) application of Fox’s equation to the T_g_ values experimentally determined by means of DSC.

The T_g_ values of the copolymers determined by DSC are given in Table 1. All DSC thermograms exhibited a single, wide glass transition (Figure 4A) and no evidence of microphase separation, which is characteristic of random copolymers. Furthermore, the T_g_ values of the poly(QMES-co-VP) copolymers are in the range between the T_g_ of the PQMES (38.0 °C) and PVP (163.4 °C) homopolymers. Figure 4B compares the T_g_’s values obtained experimentally for the copolymers with respect to those calculated using the Fox equation (theoretical T_g_). With this equation, it is possible to predict the T_g_ values of copolymer systems at any monomer composition, having known the T_g_ values of corresponding homopolymers, and assuming that both monomeric units do not interact with each other and that they contribute to the chain flexibility in an additive way [33,34,35]. Fox’s equation is expressed mathematically as follows:

$$\frac{1}{T_{g}}=\frac{W_{QMES}}{T_{g\_PQMES}}+\frac{W_{VP}}{T_{g\_PVP}}$$

where W_QMES_ and W_VP_ are the weight fractions of the QMES and VP monomers in the copolymer; T_g_PQMES_ and T_g_PVP_ are the T_g_ values (in Kelvin) obtained experimentally for PQMES and PVP, respectively; while T_g_ is the theoretical glass transition temperature that it is required to calculate for each copolymer of different composition.

In the graph of the Fox’s equation (Figure 4B) a significant fit of the experimental data is observed with respect to the theoretical ones for the copolymers with the highest composition of QMES (-51, -59, -62); therefore, it is very likely that there is a uniform distribution of both monomeric residues (VP and QMES) in the copolymer chain, which weakens the strong attractions between VP units (inter- and intra-chain) and induces weak intermolecular forces (almost negligible) that only have an additive influence on its flexibility. It is also observed that the experimental T_g_ values (T_g__exp) are lower than the theoretical ones (T_g__fox) for QMES-30 and QMES-40 (see Table 1). In the case of QMES-30, the difference between T_g__exp and T_g__fox is 9.6 °C (65.5 vs. 75.1), while in the case of QMES-40 it is 7.0 °C (57.7 vs. 64.7); this could be attributed to a “less uniform” distribution of QMES units along the copolymeric chain, characterized by having a hydrophobic end rich in QMES units, and the other end relatively hydrophilic and rich in VP units. However, the few QMES units incorporated into the hydrophilic end of QMES-30 and QMES-40 induce increased plasticity to the copolymer backbone, relative to PVP. The latter is due to the greater steric volume and flexibility of QMES units compared to VP units, which impart a lower packing density, and reduce the effective intra- or inter-chain attractions between VP units (of the dipole–dipole type) [36,37].

It is worth clarifying that the idea of copolymeric chains enriched at one end with QMES and at the other end by VP is based on the trend found in previous studies that report reactivity ratios close to zero (0) and greater than one (>1) for VP and methacrylic monomers, respectively, during free radical copolymerization of these species [38,39]. This means that the growing radicals from the methacrylic monomer prefer to homopropagate and those from VP prefer to cross-propagate. Consequently, reactions with different feed QMES molar fractions (f_QMES_) produce similar QMES molar fractions in the copolymer (F_QMES_), but only up to low-medium conversion (~40%); and as conversion increases, F_QMES_ gradually decrease, which implies that chain end-sequences of the copolymer should be enriched with the less reactive monomer, VP. Furthermore, when the feed QMES molar fraction (f_QMES_) decreases, the chain end rich in VP units (hydrophilic) is longer than the end rich in QMES units (hydrophobic) and vice versa.

### 3.4. GPC Analysis of QMES-VP Copolymers and Homopolymers

The average molecular weight (M_w_ and M_n_) and polydispersity index (M_w_/M_n_ = Ð) of the polymers measured by GPC are listed in Table 1. The molecular weight distributions showed a polydispersity index (Ð) close to two, which is usual for polymers synthesized by conventional radical polymerization (Table 1), since the disproportionation termination mechanism predominates in this reaction [40]. In the GPC chromatograms (Figure S2, see Supplementary Materials), it can be seen that all the synthesized polymers (except PVP) have a molecular weight distribution in which a broad main peak appears with a shoulder peak slightly shifted to its right. The height of the main peak decreases (relative to shoulder peak) as the molar fraction of QMES in the copolymer decreases, thus: in QMES-62, it is almost twice the shoulder peak until equal to the height of the shoulder peak in QMES-30. This result suggests that each copolymer (and also PolyQMES) might be composed not only of linear chains, but also of chains with small side branches; the latter produced by reactions of “chain transfer to the polymer” [41,42,43].

As the molar fraction of QMES in the copolymer increases the proportion of branched chains grow, and the rising in M_w_ values and reaction yield are also simultaneously observed, along with the decrease in T_g_. The T_g_ diminishes because the QMES units have a linear and flexible molecular fragment that separates the quinoline ring from the polymer backbone (i.e, spacer), and they also induce branches in the polymer chain (as mentioned above), which increases the degree of molecular disorder in the polymer. In addition, decreasing the molar fraction of VP (by increasing that of QMES) weakens the stiffness of the copolymer, taking into account that VP is a cyclic amide directly attached to the polymer chain and therefore has fewer degrees of freedom of movement and strong dipole interactions with other VP units [44]. Intra- and/or inter-catenary attractions involving sequences rich in QMES units would be weaker than those involving sequences rich in VP.

### 3.5. Thermogravimetric Analysis of QMES-VP Copolymers and Homopolymers

Table 2 shows the results of thermogravimetric analysis (TGA) of PVP, PQMES and QMES–VP copolymers (Figure S3, see Supplementary Materials). DTG thermograms for the PVP homopolymer revealed a single decomposition maximum in the temperature range between 370 and 470 °C (438.7 °C), which can be attributed to the depolymerization of the polymer backbone, along with simultaneous reactions yielding oligomers. The thermal degradation profile for the PQMES homopolymer exhibited a two-stage decomposition process. The first stage is located in the temperature range between 225 and 335 °C (287.5 °C), which corresponds to the highest weight loss of the sample with 42.9%, and can be attributed to the decomposition of the ester group that binds the quinoline ring of QMES (i.e., QOH) to the polymer backbone. The second stage of degradation (28.2% weight loss) is observed in the range of 325 to 425 °C (368.9 °C) and corresponds to the thermal decomposition of the main chain lacking QOH side groups (eliminated in the first stage). Comparing the QMES–VP copolymers with the respective homopolymers, the following conclusions can be reached: (1) the lowest decomposition temperature of all the copolymers, between 225 and 335 °C, associated with a weight loss of 33–35%, it is due to the degradation of ester bonds that attach the QMES-pending groups to the polymer; and (2) the maximum decomposition temperature of all the copolymers, which corresponds to a weight loss of approximately 33–46%, is in the range where the degradation of the main chain of both homopolymers happen (335 to 475 °C).

Therefore, it is attributed to the depolymerization of the intact VP units and the degraded methacrylic units (lacking the QOH ring) in the lower temperature stage. It is important to highlight that both degradation temperatures increase with the content of VP in the copolymer. Consequently, the incorporation of the VP units along the copolymer chain significantly improves the thermal stability of the copolymers compared to the PQMES homopolymer. All these results demonstrate the successful copolymerization between QMES and VP in a wide range of compositions.

### 3.6. Characterization of α-TOS-Loaded and Empty Poly(QMES-co-VP) Nanoparticles

On the other hand, the moderate amphiphilic nature of the poly(QMES-*co*-VP) chains, especially QMES-30 and QMES-40, induced their self-assembly in aqueous medium (in PBS, pH 7.4) during nanoprecipitation, producing stable NPs with a micellar structure (core-shell type). These polymeric chains probably have their VP-rich end oriented towards the outer surface of the micelle (shell), and their QMES-rich end oriented towards the core thereof. Table 3 shows the results of the hydrodynamic diameter (D_h_) and polydispersity index (PDI) of the NPs measured by the DLS technique. The NPs are henceforth termed according to their molar composition of QMES in the copolymer, e.g., NP-QMES-30 are those NPs obtained with the QMES-30 copolymer. The QMES-59 and QMES-62 copolymers did not form nanoparticles but, instead, aggregates that adhered instantly to the inner wall of the glass vial, which prevented their analysis by DLS.

**Figure 5:**
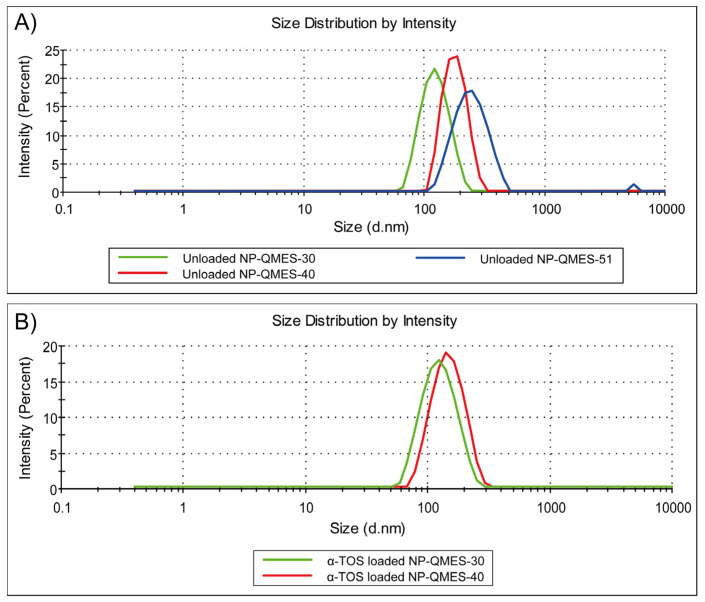
DLS size distribution curves of: (**A**) unloaded NP-QMES-30, NP-QMES-40 and NP-QMES-51. (**B**) α-TOS loaded NP-QMES-30 and NP-QMES-40.

Figure 5 shows the DLS size distribution curves of NP-QMES-30, NP-QMES-40 and NP-QMES-51. In this figure, it can be seen that the size distribution of the NP-QMES-30 and NP-QMES-40 was unimodal, while in the case of the NP-QMES-51 (with the highest PDI value, 0.331) it was bimodal. After 3 days at rest, a white precipitate was formed in the NP-QMES-51 suspension, thereupon it was discarded.

The D_h_ of newly prepared NP-QMES-30 and NP-QMES-40 without α-TOS were 118.9 and 177.5 nm, respectively, presenting low PDI values that confirm size uniformity and the absence of particle aggregates. Furthermore, the zeta potential values of NP-QMES-30 and NP-QMES-40 (electrophoretic mobility experiment), were −3.2 and −2.8 mV, respectively, indicating an almost neutral charge of the surface. This was to be expected, since both monomer units are uncharged. It is important to highlight that the NPs were stable for at least 30 days at room temperature, which was visually evidenced by the absence of precipitate.

Comparing the results of Table 3, it can be observed that the diameter of the NP-QMES-30 loaded with α-TOS is greater than that of its unloaded version (128.6 vs. 118.9 nm). This can be attributed to a higher number of polymer chains per nanoparticle, which increased the volume of the micellar core during their aggregation, in order to incorporate the α-TOS molecules. In contrast, the diameter of the NP-QMES-40 loaded with α-TOS is smaller than that of its uncharged version (148.8 vs. 177.5 nm), probably due to a better organization of the hydrophobic core induced by the encapsulated α-TOS molecules [15]. The latter and the fact of having encapsulated 13% more α-TOS than NP-QMES-30 (65% vs. 52%), suggests the additional presence of α-TOS in the shell of NP-QMES-40. In fact, the increase in the zeta potential (more negative values) of the drug loaded NPs with respect to the empty ones (see Table 3), indicates that possibly the polar carboxylic groups of α-TOS are at least partially oriented towards the hydrophilic shell (and its chromanol fragment oriented towards the core), giving them greater stability in the aqueous medium [15]. This zeta potential result suggests that the shell of NP-QMES-30 also contains α-TOS, although in less quantity than in the shell of NP-QMES-40.

**Figure 6:**
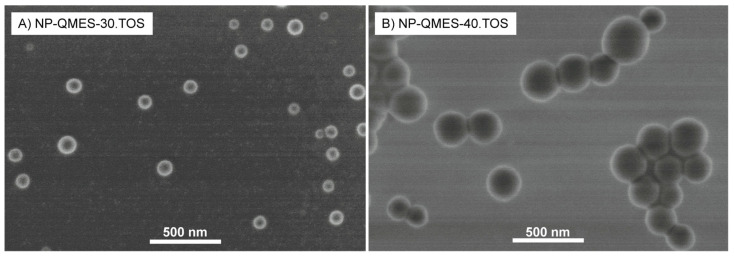
SEM micrographs of α-TOS loaded nanoparticles dried from aqueous solution at room temperature. Scale bars: 500 nm.

SEM images of α-TOS loaded NP-QMES-30 and NP-QMES-40 confirmed a spherical morphology (Figure 6A,B) and a considerably larger diameter for NP-QMES-40. However, contrary to expectations, in the case of α-TOS loaded NP-QMES-40, the diameter for dry NPs measured by SEM (Figure 6B) was higher than the hydrodynamic diameter measured by DLS (Table 3, Figure 5B), and only in the SEM images was high aggregation tendency is observed. This anomalous behavior of α-TOS loaded NP-QMES-40 in comparison with NP-QMES-30 (Figure 6A) is probably due to the higher hydrophobic/hydrophilic ratio of their copolymer chains, which along with the drying process required in the sample preparation for SEM, caused the coalescence of the nanoparticles [45,46]. For biomedical applications in aqueous environments, the hydrodynamic diameter (DLS) is more relevant than the diameter measured in dry state from SEM.

As both systems of NPs have hydrodynamic diameters between 100 and 200 nm, they can experience the EPR (enhanced permeability and retention) effect in cancerous tumors, without being eliminated by the reticuloendothelial system (which occurs to NPs greater than 200 nm in size) or by cellular endocytosis (which occurs to NPs less than 100 nm in size) [13,15]. For all of the above, these NPs were chosen for cytotoxicity bioassays on cancer cells.

### 3.7. Uptake and Co-Localization of c6-Loaded NP

**Figure 7:**
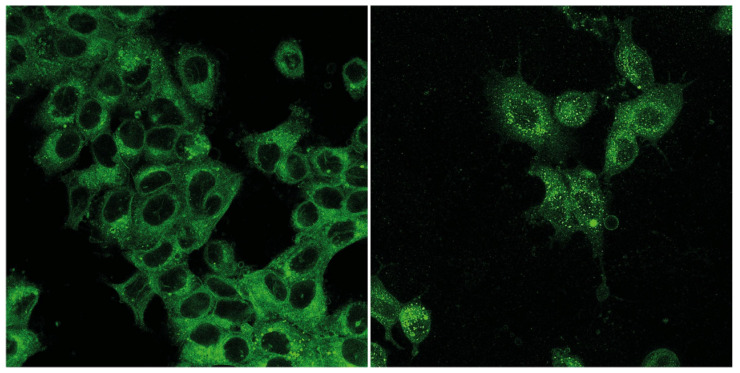
Confocal micrographs of cellular uptake of c6-loaded NP-QMES-30 in MCF-7 cells.

The hydrodynamic diameters (D_h_) of NP-QMES-30 and NP-QMES-40 loaded with the fluorescent probe coumarin-6 were 124.4 and 247.3 nm, respectively, presenting nearly neutral charge on their surface and low PDI values (Table 3). In addition, DLS size distribution curves were unimodal (Figure S4, see Supplementary Materials), suggesting uniformity of the particle size without signs of aggregation. Only the cellular internalization of c6 loaded NP-QMES-30 could be studied, since c6 loaded NP-QMES-40 were retained in the sterilizing syringe filter due to them having diameters greater than 200 nm (~247 nm, Table 3). In Figure 7, it is observed that the c6 loaded NP-QMES-30 (green fluorescence color) crossed the cell membrane and accumulated in the cytoplasm of MCF-7 cells (around the nucleus), after being in contact with the cells overnight. Endocytosis is the most likely mechanism of internalization of these NPs, due to their size ranging from 100 to 200 nm [47].

**Figure 8:**
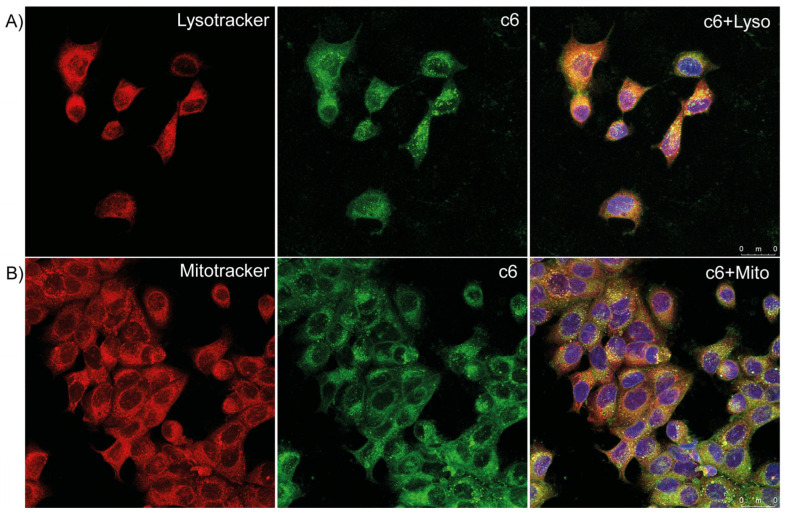
Co-localization of c6 loaded NP-QMES-30 into (**A**) the lysosomes and (**B**) the mitochondria of MCF-7 cells by confocal microscopy.

Figure 8A,B with their three confocal micrographs each, from left to right, show successively: (1) lysosomes (“Lysotracker”) and mitochondria (“Mitotracker”), in red; (2) c6 loaded NPs inside of cytoplasm (“c6”), in green; and (3) c6 loaded NPs specifically located inside of lysosomes (“c6+Lyso”) and mitochondria (“c6+Mito”), in orange. Orange fluorescence is the result of the combination of green and red fluorescent images, confirming the colocalization of c6-loaded NPs in both the lysosomes and mitochondria of MCF-7 cancer cells. However, colocalization seems to be greater in lysosomes compared to mitochondria.

### 3.8. Cytotoxic Activity of Poly(QMES-co-VP) Nanoparticles

**Figure 9:**
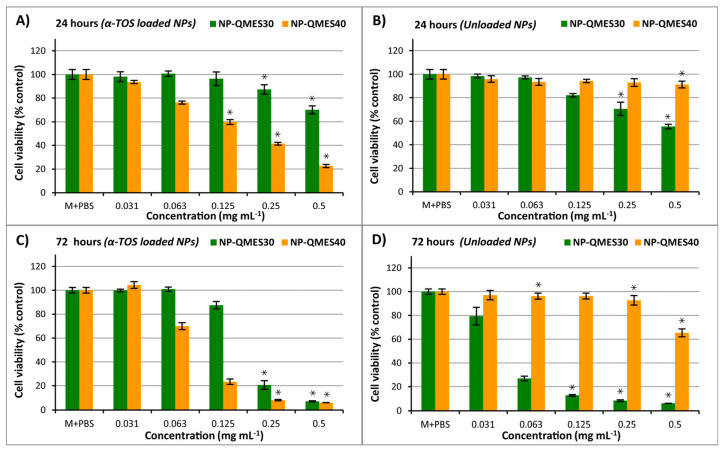
MFC-7 cells viability exposed to different concentrations of NP-QMES-30 (green bars) or NP-QMES-40 (orange bars): loaded with α-TOS and 24 h (**A**) or 72 h (**C**) of treatment; or “unloaded” and 24 h (**B**) or 72 h (**D**) of treatment. The diagrams include the mean, the standard deviation (*n* = 8), and Kruskal–Wallis test or ANOVA results. The asterisk (*) denotes a statistically significant difference (*p* < 0.05) compared to the control (M + PBS).

**Figure 10:**
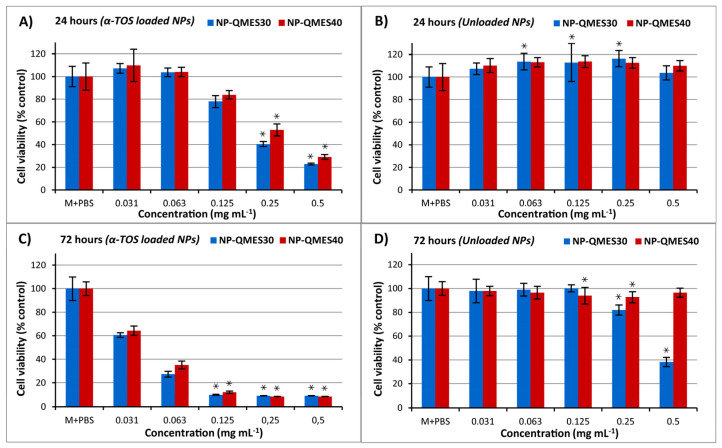
HUVEC cells viability exposed to different concentrations of NP-QMES-30 (blue bars) or NP-QMES-40 (red bars): loaded with α-TOS and 24 h (**A**) or 72 h (**C**) of treatment; or “unloaded” and 24 h (**B**) or 72 h (**D**) of treatment. The diagrams include the mean, the standard deviation (*n* = 6), and Kruskal–Wallis test or ANOVA results. The asterisk (*) denotes a statistically significant difference (*p* < 0.05) compared to the control (M + PBS).

The cytotoxicity of the NP-QMES-30 and NP-QMES-40 (empty or α-TOS loaded) was evaluated by means of AlamarBlue assays, both in cancer MCF-7 cells and healthy HUVEC cells, and the results are shown in Figure 9 and Figure 10, respectively. It should be noted that QOH and α-TOS were insoluble in the cell culture medium (aqueous), which prevented the comparison of their cytotoxic effect against that of empty or loaded NPs.

In the case of empty NP-QMES-30, it can be observed that they are cytotoxic (i.e., <70% viability) for MCF-7 cells at both 24 (Figure 9B) and 72 (Figure 9D) hours of treatment, but this effect is more marked at 72 h with an IC_50_ value of 0.043 mg mL^−1^. While empty NP-QMES-40 only become cytotoxic after 72 h of treatment with a concentration of 0.5 mg mL^−1^ (~65% viability, Figure 9D). The lower cytotoxic effect of NP-QMES-40 compared to NP-QMES-30 (in their empty versions) is probably due to the former having: (1) a larger size (177.5 vs. 118.9 nm) that makes it difficult for them to enter cells, and (2) a lower concentration compared to that initially established (in its preparation), as a consequence of the filtration–sterilization of the NP suspensions (with 0.2 micron filters), resulting in the removal of a part of the largest NPs.

In Figure 10B,D, it is observed that empty NP-QMES-30 (blue bars) and NP-QMES-40 (red bars) are not cytotoxic for healthy HUVEC cells at 24 h, while at 72 h, NP-QMES-30 reduces viability only to the highest concentration tested, i.e., 0.5 mg mL^−1^. Therefore, a selective cytotoxicity of NP-QMES-30 against cancer cells (MCF-7), compared to normal cells (HUVEC) is demonstrated (compare Figure 9D green bar vs. Figure 10D blue bar). Assuming that NP-QMES-30, in contact with cells, releases QOH, then the selectivity of NPs towards MCF-7 cells could be related to the mechanism of action of QOH, but this is unknown to date. However, it is known that its chemical analogue, QOH acrylate, interacts with Fe^3+^ ions inhibiting the growth of some bacteria that have inefficient cellular iron uptake mechanisms [29]. The latter suggests that QOH, like its chemical analogue, could act in a similar way to an iron chelating agent, depleting the source of ferric ion required (as a nutrient) for the growth of both bacteria and human cancer cells. The culture medium used in this research is the source of ferric ion for the cells. It should be noted that the therapeutic potential of iron chelators as antitumor agents is widely known, and is based on the fact that the rapidly dividing cancer cells require more iron than normal healthy cells and are particularly sensitive to the depletion of cellular iron [48,49,50]. The increased requirement of iron in cancer cells is due to the fact that they over-express proteins and enzymes that contain iron in their structure, such as: ribonucleotide reductase, required for DNA synthesis; ferritin, used for iron storage; and the transferrin receptor involved in endocytosis of the transferrin–iron complex [48]. Therefore, the fact that empty NP-QMES-30 were cytotoxic for MCF-7 (cancer) cells and innocuous for HUVEC (healthy) cells may be related to the iron chelation mechanisms mentioned above.

On the other hand, since the empty NP-QMES-40 were innocuous for MCF-7 cells at the times tested (Figure 9B,D orange bars), it was decided to load them with α-TOS as a strategy to increase cytotoxicity against these cancer cells. The NP-QMES-40 loaded with α-TOS were cytotoxic for MCF-7 cells, both at 24 h and 72 h of treatment (Figure 9A,C, orange bars), with IC_50_ values of 0.156 mg mL^−1^ and 0.076 mg mL^−1^, respectively, demonstrating a progressive release of α-TOS. The higher cytotoxicity of the loaded NP-QMES-40 (Figure 9A,C), compared to the empty ones (Figure 9B,D), can be explained by the smaller diameter of the former (148.8 vs. 177.5 nm) as a consequence of the better organization of the hydrophobic core, induced by the encapsulated α-TOS molecules [14]. In addition to enhancing cytotoxicity against cancer cells, a good encapsulation efficiency of ~65% was also achieved, which make it into a potential candidate for being a drug delivery vehicle. However, the NP-QMES-40 loaded with α-TOS were also cytotoxic for healthy HUVEC cells (confirming the lack of selectivity towards MCF-7 cells; see Figure 10A,C, red bars), probably due to an excess of encapsulated α-TOS, which could be optimized in later studies, in order to achieve the desired selectivity.

## 4. Conclusions

In this study, QMES monomer (QOH analogue) was successfully copolymerized with N-vinyl-pyrrolidone (VP) by conventional radical polymerization. The amphiphilic nature of the obtained QMES-30 and QMES-40 copolymers allowed them to self-assemble in an aqueous medium, forming nanoparticles (NPs) with optimal sizes and properties for their application in the treatment of cancer.

In the cell viability assay, empty NP-QMES-30 showed selective cytotoxicity towards MCF-7 cancer cells (IC_50_ = 0.043 mg mL^−1^) relative to healthy HUVEC cells; however, when they were loaded with α-TOS (with an encapsulation efficiency of 52%) they lost selectivity and were toxic for both cell types. Consequently, empty NP-QMES-30 became a promising polymeric prodrug candidate for cancer treatment.

In contrast, empty NP-QMES-40 were innocuous to both cancer and healthy cells, while its α-TOS-loaded version (with an encapsulation efficiency of 65%) was cytotoxic to both cell types. This last result suggests that NP-QMES-40 can be a good nanocarrier of α-TOS (or other hydrophobic drugs) if the incorporated amount of this drug in the nanoparticles is optimized.

## Figures and Tables

**Table 1:** Feed (f) and copolymer (F) QMES molar fractions, copolymer (W) QMES weight fractions, T_g_’s, reaction yields, molecular weights (M_w_ and M_n_) and polydispersity indices (M_w_/M_n_ = Ð) for the synthesized poly(QMES-co-VP).

Copolymer	f_QMES_	F_QMES_	W_QMES_	Yield (%)	T_g_ * (°C)	T_g_Fox_^≠^ (°C)	M_w_ (KDa)	M_n_ (KDa)	Ð
PVP	0	0	0	55.2	163.4	163.4	18.6	13.1	1.42
QMES-30	0.20	0.30	0.63	56.2	65.5	75.1	18.7	9.50	1.96
QMES-40	0.30	0.40	0.73	61.6	57.7	64.7	23.7	11.4	2.09
QMES-51	0.50	0.51	0.80	68.9	56.3	56.5	29.1	13.4	2.18
QMES-59	0.70	0.59	0.85	76.4	52.4	52.0	31.2	13.9	2.25
QMES-62	0.80	0.62	0.87	84.4	45.9	50.5	32.9	14.1	2.34
PQMES	1	1	1	82.2	38.0	38.0	26.7	13.3	2.01

*: Experimental T_g_ values of copolymers; ^≠^: Theoretical T_g_ values estimated by Fox’s equation.

**Table 2:** Results of thermogravimetric analysis (TGA) of the synthesized PVP, PQMES and the poly(QMES-co-VP).

Copolymer	T_g_A1 (°C)	ΔW1	T_g_A2 (°C)	ΔW2
PVP	---	---	438.7	89.8
QMES-30	302.8	32.8	400.8	46.0
QMES-40	303.4	33.9	392.9	37.8
QMES-51	305.2	34.8	388.5	38.3
QMES-59	294.2	34.3	385.1	36.0
QMES-62	291	32.9	370.6	33.4
PQMES	287.5	42.9	368.9	28.2

**Table 3:** Hydrodynamic diameter (D_h_, by intensity), polydispersity index (PDI), zeta potential (ZP), and α-TOS or c6 weight percentage of poly(QMES-co-VP) NPs of different copolymer QMES molar fractions (F_QMES_) prepared at 1 mg mL^−1^ in PBS by nanoprecipitation. Data were obtained by DLS with Zetasizer Lab equipment from Malvern.

Sample	F_QMES_	[NPs] (mg mL^−1^)	α-TOS (%)	c6 (%)	D_h_ (nm)	PDI	ZP (mV)	EE (%)	* LE (mg mg^−1^)
NP-QMES-30	0.30	1	0	0	118.9 ± 2.5	0.056 ± 0.017	−3.2 ± 0.5	---	---
NP-QMES-30.TOS	0.30	1	10	0	128.6 ± 5.8	0.063 ± 0.020	−15.3 ± 1.5	52 ± 3	0.052
NP-QMES-30.c6	0.30	1	0	1	124.4 ± 2.7	0.120 ± 0.020	2.5 ± 0.2	---	---
NP-QMES-40	0.40	1	0	0	177.5 ± 3.4	0.031 ± 0.010	−2.8 ± 0.4	---	---
NP-QMES-40.TOS	0.40	1	10	0	148.8 ± 8.6	0.060 ± 0.015	−17.6 ± 3.2	65 ± 5	0.065
NP-QMES-40.c6	0.40	1	0	1	247.3 ± 2.7	0.191 ± 0.012	2.2 ± 0.5	---	---
NP-QMES-51	0.51	1	0	0	312.2 ± 5.2	0.331 ± 0.050	---	---	---

*: Loading efficiency, i.e., the amount of α-TOS loaded into the nanocarrier, and expressed in mg of α-TOS per mg of copolymer.

## Data Availability

The data presented in this study are available on request from the corresponding author.

## Supplementary Materials

The following supporting information can be downloaded at: https://www.mdpi.com/article/10.3390/polym15224342/s1, Figure S1: UV spectra of: α-TOS loaded and unloaded NP-QMES-30; Figure S2: GPC chromatograms of the synthesized poly(QMES-co-VP) copolymers; Figure S3: Results of thermogravimetric analysis (TGA) of PVP, PQMES and QMES-VP copolymers; Figure S4: DLS size distribution curves of: c6 loaded NP-QMES-30 (D_h_ = 124.4 ± 2.7; PDI = 0.120) and c6 loaded NP-QMES-40 (D_h_ = 247.3 ± 2.7; PDI = 0.191).
