# Supplementary material for: Nanocarrier of α-Tocopheryl Succinate Based on a Copolymer Derivative of (4,7-dichloroquinolin-2-yl)methanol and Its Cytotoxicity against a Breast Cancer Cell Line
Source: Polymers (Basel). 2023 Nov 7;15(22):4342. doi: 10.3390/polym15224342 (PMC10674486; doi:10.3390/polym15224342)
Supplement: Supplementary file 1 [file polymers-15-04342-s001.zip › polymers-2516643-supplementary.pdf]

polymers

Supporting material

Nanocarrier of α-Tocopheryl Succinate based on a Copolymer Derivative of (4,7-dichloroquinolin-2-yl)methanol and its Cytotoxicity against a Breast Cancer Cell line

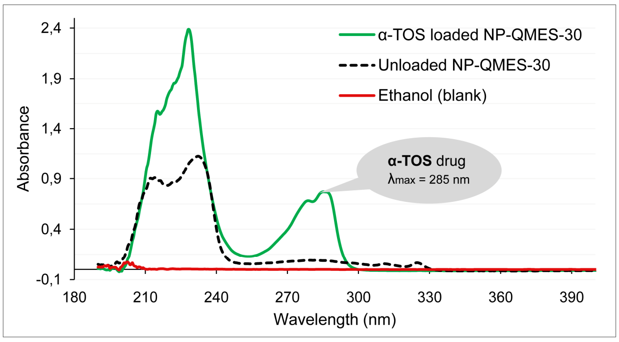

Figure S1. UV spectra of: α-TOS loaded and unloaded NP-QMES-30.

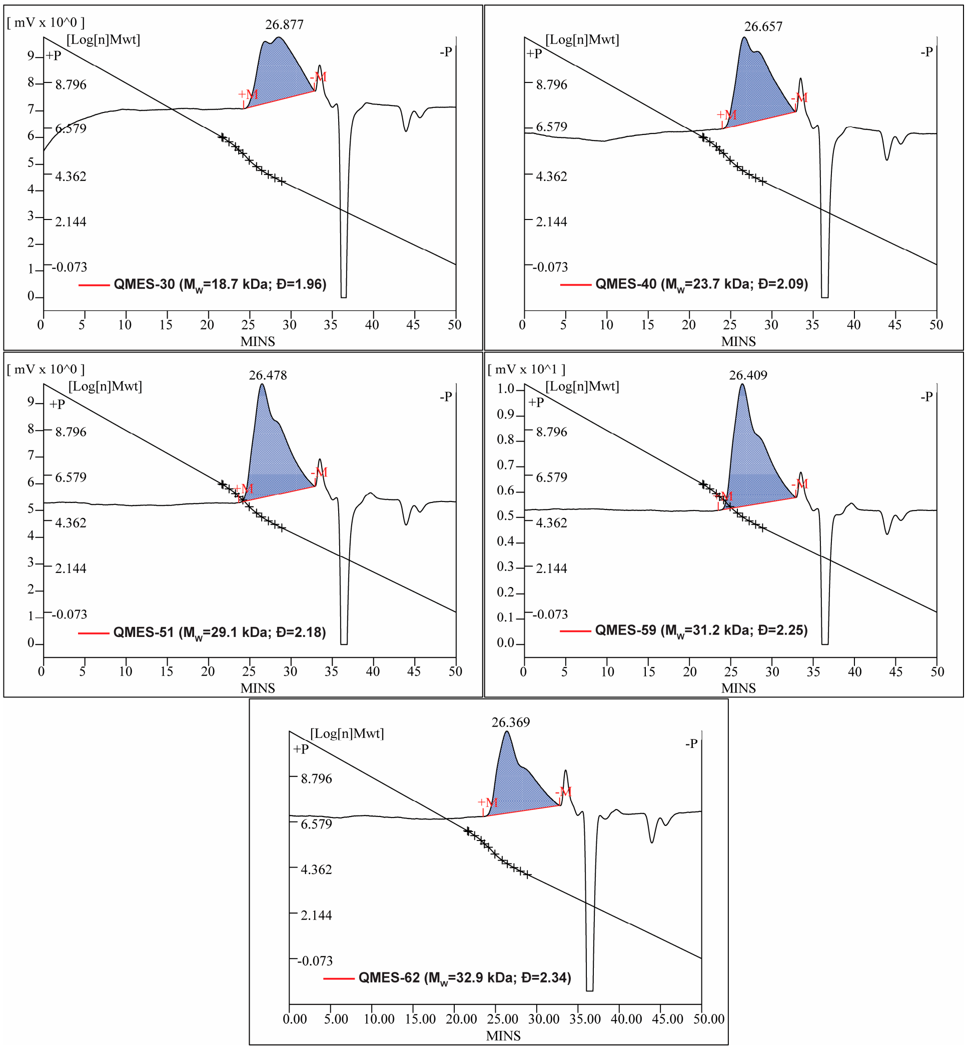

Figure S2. GPC chromatograms of the synthesized poly (QMES-co-VP) copolymers.

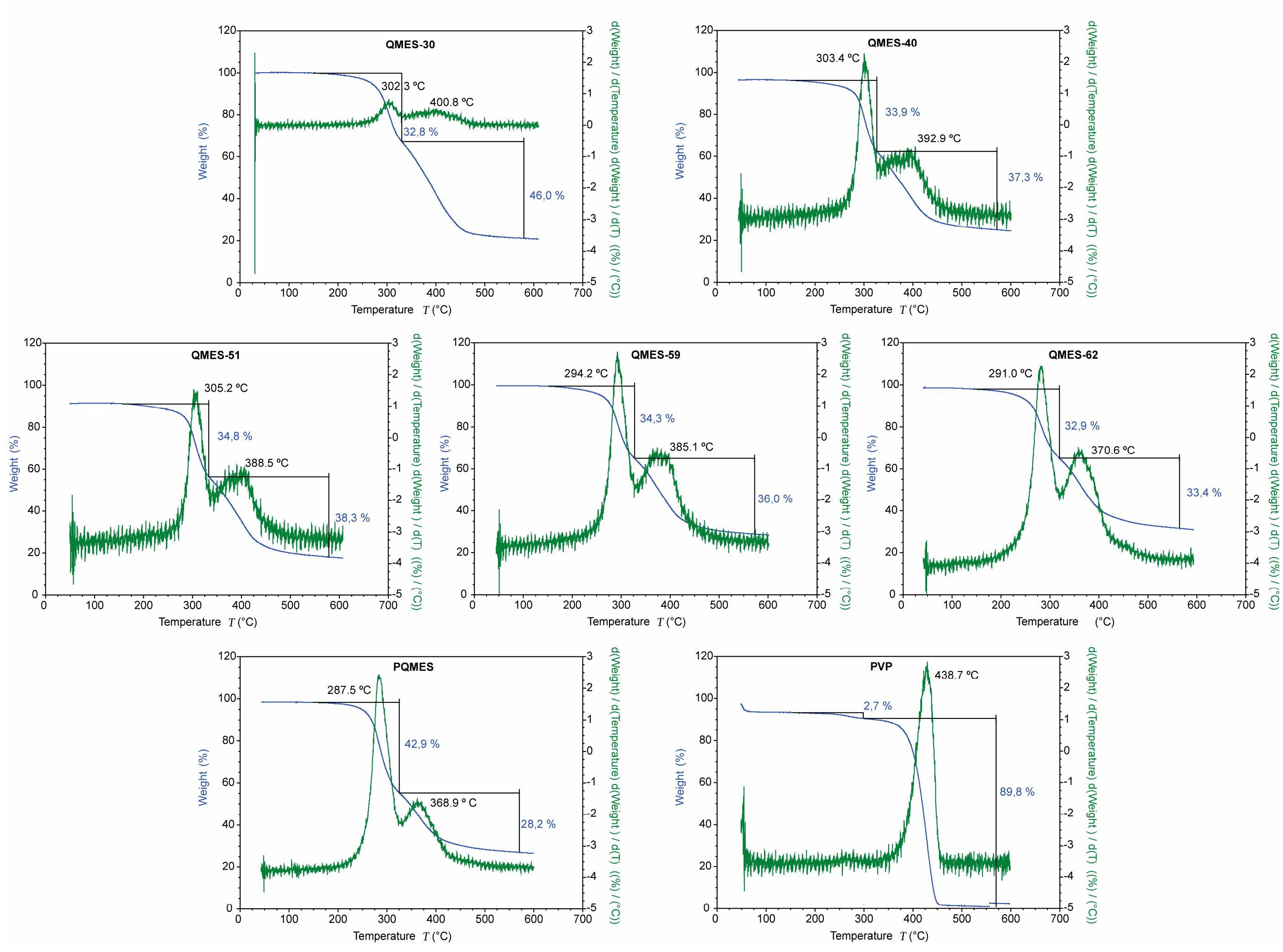

Figure S3. Results of thermogravimetric analysis (TGA) of PVP, PQMES and QMES-VP copolymers.

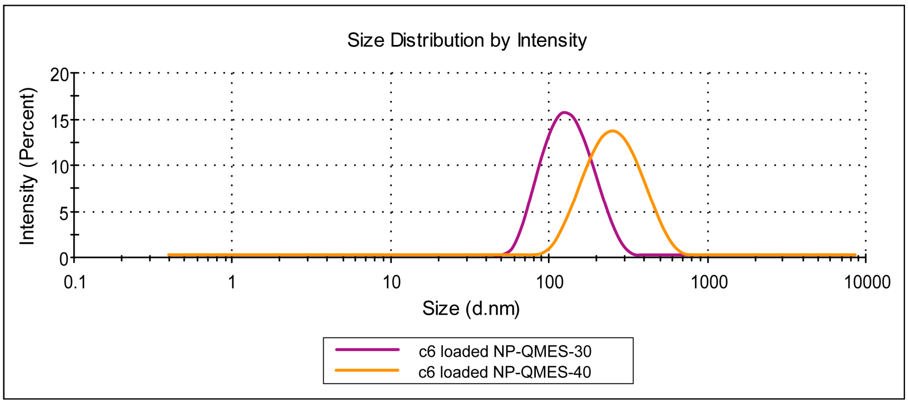

Figure S4. DLS size distribution curves of: c6 loaded NP-QMES-30 (D_h = 124.4 ± 2.7; PDI = 0.120) and c6 loaded NP-QMES-40 (D_h = 247.3 ± 2.7; PDI = 0.191).
